# Transcriptome analysis of *Phytophthora cactorum* infecting strawberry identified RXLR effectors that induce cell death when transiently expressed in *Nicotiana benthamiana*

**DOI:** 10.3389/fpls.2024.1379970

**Published:** 2024-05-24

**Authors:** Bikal Ghimire, Anupam Gogoi, Mandeep Poudel, Arne Stensvand, May Bente Brurberg

**Affiliations:** ^1^ Department of Plant Sciences, Faculty of Biosciences (BIOVIT), Norwegian University of Life Sciences (NMBU), Ås, Norway; ^2^ Division of Biotechnology and Plant Health, Norwegian Institute of Bioeconomy Research (NIBIO), Ås, Norway

**Keywords:** oomycete, RNA-Seq, agroinfiltration, disease resistance response, host-pathogen interaction

## Abstract

*Phytophthora cactorum* is a plant pathogenic oomycete that causes crown rot in strawberry leading to significant economic losses every year. To invade the host, *P. cactorum* secretes an arsenal of effectors that can manipulate host physiology and impair its defense system promoting infection. A transcriptome analysis was conducted on a susceptible wild strawberry genotype (*Fragaria vesca*) 48 hours post inoculation with *P. cactorum* to identify effectors expressed during the early infection stage. The analysis revealed 4,668 *P. cactorum* genes expressed during infection of *F. vesca*. A total of 539 secreted proteins encoded by transcripts were identified, including 120 carbohydrate-active enzymes, 40 RXLRs, 23 proteolytic enzymes, nine elicitins, seven cysteine rich proteins, seven necrosis inducing proteins and 216 hypothetical proteins with unknown function. Twenty of the 40 RXLR effector candidates were transiently expressed in *Nicotiana benthamiana* using agroinfiltration and five previously unreported RXLR effector genes *(Pc741, Pc8318, Pc10890, Pc20813*, and *Pc22290)* triggered cell death when transiently expressed. The identified cell death inducing RXLR effectors showed 31–66% identity to known RXLR effectors in different *Phytophthora* species having roles in pathogenicity including both activation and suppression of defense response in the host. Furthermore, homology analysis revealed that these cell death inducing RXLR effectors were highly conserved (82 - 100% identity) across 23 different strains of *P. cactorum* originating from apple or strawberry.

## 1 Introduction

*Phytophthora cactorum* is a devastating soil-borne oomycete pathogen that infects more than 200 plant species from 154 genera, including some of the most valuable horticultural plants such as apple, pear, and strawberry (Erwin and Ribeiro, 1996; Hantula et al., 2000; Sanchez et al., 2019; Chen et al., 2023). The pathogen can persist as resting oospores in soil for many years, even without a host plant, and during extreme environmental conditions. *Phytophthora cactorum* is challenging to control even with the use of chemicals due to oomyceticide (fungicide) resistance and the inefficiency of chemicals to different life stages of the pathogen (Marin et al., 2021; Ali et al., 2022). In the strawberry host, *P. cactorum* causes crown rot and leather rot, limiting the plant growth and quality of strawberry fruits, respectively, resulting in significant economic losses worldwide (Ellis and Grove, 1983; Stensvand et al., 1999). Crown rot symptoms include brown necrotic lesions in the rhizome (crown), which in severe cases result in wilting of the whole plant, whereas the leather rot affects the strawberry fruits, imparting an off-flavor taste and a pungent smell (Maas, 1998).

To establish an infection in the host plant, *P. cactorum* secretes an arsenal of effector proteins with diverse functions (Armitage et al., 2018; Gogoi et al., 2023b). These effectors are localized in the plant apoplastic spaces (apoplastic effectors) or translocate into the plant cell cytoplasm and diverse subcellular locations (cytoplasmic effectors) to enhance host colonization (Wang S. et al., 2019; Boevink et al., 2020). Apoplastic effectors include cysteine rich proteins, different cell wall degrading enzymes, elicitins, enzyme inhibitors, lipases, necrosis inducing proteins, phytotoxins, and proteolytic enzymes (proteases, peptidases). The most well studied group of cytoplasmic effectors from plant pathogenic oomycetes are the RXLRs (Arginine-any amino acid-Leucine-Arginine) and Crinklers (CRNs for crinkling and necrosis) (Kamoun, 2006; Hardham and Cahill, 2010; Wang Y. et al., 2019). The RXLR effectors have a conserved N-terminal RXLR amino acid motif often linked with an EER motif that mediates the translocation of the effector proteins into the host plant cells (Rehmany et al., 2005; Bhattacharjee et al., 2006; Whisson et al., 2007; Liu et al., 2019). Once inside the host cell, RXLRs can function both as activators of defense or suppressors of plant immunity (Birch et al., 2008; Oh et al., 2010; Anderson et al., 2015).

Plants possess a two layered immune system that responds to invading pathogens and impedes their growth. The first layer of immunity, known as pattern-triggered immunity (PTI), is activated when specific epitopes, called microbe- or pathogen associated molecular patterns (MAMPs/PAMPs), are recognized by the plant’s pattern recognition receptors (PRRs) (Medzhitov and Janeway, 1997; Jones and Dangl, 2006). The second layer of immunity, referred to as effector-triggered immunity (ETI), involves host resistance proteins that interact either directly or indirectly with specific effectors secreted by the pathogen, activating several defense signaling pathways (Jones and Dangl, 2006; Dodds and Rathjen, 2010; Dou and Zhou, 2012; Giraldo and Valent, 2013). Both PTI and ETI activation result in transcriptional reprogramming of the defense-related genes, which lead to the production of reactive oxygen species (ROS), secondary metabolites, hydrolytic enzymes, phytohormones and pathogenesis-related proteins (Tsuda and Katagiri, 2010; Naveed et al., 2020). The activation of defense responses in the host plant via PTI and ETI can also initiate a hypersensitive response or localized programmed cell death that can restrict growth of biotrophic and hemibiotrophic pathogens including *Phytophthora* species (Naveed et al., 2020).

Identifying and studying effector genes is crucial for understanding their roles in the interaction with host plants. Previous transcriptomic studies of different life stages of *P. cactorum* (mycelium, sporangia, zoospores, cysts, germinating cysts), and during infection of *Nicotiana benthamiana* and strawberry have identified several candidate effector genes (Chen et al., 2014, 2018; Nellist et al., 2021). Furthermore, *in silico* analyses of the sequenced genomes of *P. cactorum* have predicted hundreds of effector genes including RXLRs and CRNs (Armitage et al., 2018; Yang et al., 2018; Nellist et al., 2021; Gogoi et al., 2023b). To uncover the effectors produced by *P. cactorum* during the early and important phase of strawberry infection, the transcriptome was studied 48-hours post-inoculation of the rhizome (crown) of the susceptible *Fragaria vesca* genotype NCGR1218. Twenty candidate RXLR effector genes identified in the transcriptome study were transiently expressed in *N. benthamiana* leaves to examine potential cell death inducing responses.

## 2 Materials and methods

### 2.1 Plant material and *Phytophthora cactorum*

Diploid strawberry *Fragaria vesca* genotype NCGR1218 (susceptible to *Phytophthora cactorum*) was clonally propagated from runners and maintained in a greenhouse with a 16-hour photoperiod at 18°C. *Nicotiana benthamiana* plants were grown from seeds and were kept in a growth chamber at 21°C with a 16-hour photoperiod. *Phytophthora cactorum* strain 10300 previously isolated from a crown rot infected strawberry plant (*Fragaria* × *ananassa*) (Armitage et al., 2018) was routinely cultured on vegetable juice (V8) agar plates (Ferguson and Jeffers, 1999) at room temperature (~21°C) in the dark. A zoospore suspension was prepared from one-week old culture plates as described by Eikemo et al. (2000). The zoospores released from sporangia were counted using a hemocytometer and the concentration was adjusted to 2 × 10^5^ zoospores/ml for inoculation of strawberry plants. The plants were gently wounded in the rhizome (crown) with a sterile scalpel and inoculated with 2 ml of the zoospore suspension or water (mock/control). Four biological replicates, with each replicate consisting of four individual plants were used for the inoculation experiment as well as the control treatment with water. The rhizome samples were harvested 48 hours after inoculation, flash frozen in liquid nitrogen and stored at -80°C until RNA isolation. The 48 hour time point represents the early infection stage of *P. cactorum* based on a temporal expression study of defense related genes in the resistant and susceptible *F. vesca* genotypes as previously described (Toljamo et al., 2016; Chen et al., 2016a; Gogoi et al., 2023a). No visible symptoms were observed at the time of harvest. Some additional plants, both zoospore inoculated and water control, were kept up to four weeks post inoculation to study the disease progression. Wilting and necrotic lesions were observed in the *P. cactorum* inoculated plants after two weeks while no symptoms were observed in the control plants (Gogoi et al., 2023a).

### 2.2 RNA extraction, transcriptome sequencing and analysis

Total RNA was extracted from the inoculated strawberry rhizomes using the Spectrum™ Plant Total RNA Kit (Sigma-Aldrich, USA) according to the manufacturer’s instructions. On-column DNase digestion was performed for 30 minutes on the isolated RNA to remove traces of genomic DNA contamination (Sigma-Aldrich, USA), as described by Gogoi et al. (2023a). Sequencing libraries were prepared using the TruSeq™ stranded total RNA library prep Kit (Illumina), and sequencing was performed using four lanes on an Illumina HiSeq 3/4000 System (2 × 150 bp) at the Norwegian Sequencing Centre, Oslo, Norway. An average of 54.8 million high-quality trimmed reads (SD 5.3 million) were obtained from the four replicates in this study. Transcriptome assembly and expression analysis were carried out as previously described (Gogoi et al., 2023a). Briefly, the transcripts were *de novo* assembled instead of mapping to the reference genome (*P. cactorum, assembly ASM1686465v1*), to recover both *F. vesca* and *P. cactorum* transcripts. Transcripts were quantified using the pseudo-alignment method Kallisto (Bray et al., 2016), and the normalization of the transcript counts was performed in the CLC genomic workbench v11.01 (Qiagen, Aarhus, Denmark) using the transcripts per million (TPM) method (Wagner et al., 2012). The longest transcript isoforms obtained from the *de novo* assembly were assigned a *P. cactorum* gene ID using BLASTN against the sequenced genome of the *P. cactorum* strain 10300 (GCA_003287315.1_Pcac_10300_v1_cds_from_genomic), with an expectation value e < 10^–10^ as a threshold. Transcripts having ≥ 99% identity to the *P. cactorum* 10300 genome were selected for the downstream analysis. The transcripts were annotated using Blast2GO v5.0 (Götz et al., 2008). The full-length sequences of the proteins encoded by the genes were retrieved using NCBI protein sequences (GCA_003287315.1_Pcac_10300_v1_protein), and these were used for further analysis. The full-length protein sequences encoded by transcripts were analyzed using the STRING database V11.5 (Szklarczyk et al., 2021), with gene ontology (GO) classification for functional annotation and the Kyoto encyclopedia of genes and genomes (KEGG) to predict biological pathways.

### 2.3 Secretome prediction and *in-silico* functional analysis of effector proteins

Effector proteins must be secreted in order to reach their cellular targets at the intercellular interface between the plant and pathogen or inside the host cell (Torto et al., 2003), and therefore the proteins encoded by transcripts were analyzed for signal peptides using SignalP5 (Armenteros et al., 2019b). The proteins predicted to have a signal peptide were further analyzed for transmembrane domains using Phobius (Käll et al., 2004), mitochondrial transit peptides using TargetP2.0 (Armenteros et al., 2019a), and endoplasmic reticulum retention signals (KDEL/HDEL motif) (Stornaiuolo et al., 2003) using PROSITE-Scan (de Castro et al., 2006). All proteins with a signal peptide in their N-termini with no more than one transmembrane domain and no mitochondrial transit peptide or endoplasmic reticulum retention motif were considered to be secreted proteins of *P. cactorum*.

The predicted secreted proteins were examined for carbohydrate active enzymes (CAZymes) using HMMER: dbCAN3-sub tool (e-value < 1e-15; coverage > 0.35) in the dbCAN3 meta server (Huang et al., 2018), while CRN and RXLR effectors were identified using the *effectR* package in R v4.2.0 (Tabima and Grünwald, 2019).

Additionally, all proteins characterized as secreted were searched against the pathogen-host interaction database (PHI-base) 4.14 using BLASTP (e-value <1e-5; ≥50% query coverage; bit score ≥50; identity ≥30%). The PHI-base comprises experimentally validated genes associated with pathogen virulence, which can aid in uncovering the role of secreted proteins in pathogenicity or disease development (Urban et al., 2022). The PHI-phenotypes ‘increased virulence’, ‘lethal’, ‘loss of pathogenicity’, and ‘reduced virulence’ are produced as a result of a mutation or altered expression of a specific gene in the pathogen, while the PHI-phenotype ‘plant avirulence determinant’ represents the effector gene required for the recognition of a pathogen in resistant hosts (Urban et al., 2022).

### 2.4 Protein homology and substitution rates analyses of the RXLR effectors

The predicted protein structures of 39 of the 40 RXLR effectors detected in this study (PC110_g6139 was unavailable) were downloaded as PDB files from the AlphaFold Protein Structure Database (Varadi et al., 2022). The predicted structure of the RXLR effectors were aligned using template modeling (TM)-align (Zhang and Skolnick, 2005) and a TM-score matrix was constructed for topological structural similarity assessment of the protein structures. Protein pairs with a TM-score > 0.5 are considered to share the same global fold, while those with a TM-score < 0.5 do not (Xu and Zhang, 2010). Additionally, homologs of the five RXLR effectors that induced cell death in *N. benthamiana* were identified from 23 different strains (**Supplementary Material S5**) of *P. cactorum* using OrthoFinder v2.4.0 (Emms and Kelly, 2019). The protein sequences of the identified homologs for each RXLR were aligned using Clustal Omega (Madeira et al., 2022) and phylograms were constructed using the neighbor-joining method and visualized using PRESTO (http://www.atgc-montpellier.fr/presto).

The rate of synonymous (α) and nonsynonymous (β) substitutions in the homologs of cell death inducing RXLR effectors were calculated for the four RXLRs that had variation within available sequences using FUBAR (Fast, Unconstrained Bayesian AppRoximation) from the HyPhy software v2.5.29 (MP) (Murrell et al., 2013; Kosakovsky Pond et al., 2020). Amino acids sites with β > α and posterior probability > 0.9 were considered under positive (diversifying) selection, whereas β < α (with posterior probability > 0.9) were considered under negative (purifying) selection.

### 2.5 Cloning of *P. cactorum* RXLR effector genes and their transient expression in *Nicotiana benthamiana*

To understand the role of RXLR effectors in inducing plant immunity, *RXLR* candidate genes were cloned in a plant expression vector and were transiently expressed in *N. benthamiana* leaves. For cloning, cDNA was used as a template for amplification of the target *RXLR* genes. Briefly, one microgram of total RNA isolated from the infected rhizome samples of the susceptible strawberry genotype NCGR1218 was used for cDNA synthesis using the iScript™ cDNA Synthesis Kit (Bio-Rad, USA). The full-length genes without the signal peptide region were amplified in a 50 µl PCR reaction mix with Phusion^®^ High-Fidelity DNA Polymerase (5 U/µl) (New England Biolabs, USA) using gene-specific primers (**Supplementary Table S1**). PCR was performed in the T100 Thermal Cycler (BioRad, USA) with an initial denaturation at 95°C for five minutes followed by 35 cycles of denaturation at 95°C for 30 seconds, annealing at 60°C for 30 seconds, and polymerization at 72°C for 1 minute with a final extension of 12 minutes at 72°C. The amplified products were gel purified using QIAquick Gel Extraction Kit (Qiagen, USA), cloned into the Gateway entry vector pDONR™/Zeo (Thermo Fisher Scientific, USA) by BP recombination, and subsequently moved into the plant expression vector pK7WG2 (Karimi et al., 2002) through an LR recombination reaction (Thermo Fisher Scientific, USA). The constructs were transformed into chemically competent *Escherichia coli* DH5α cells (Library Efficiency™ DH5α competent cells, Invitrogen, USA) using the heat-shock transformation method (Froger and Hall, 2007). Transformants were selected using Luria-Bertani agar (LA) plates containing 50 µg/ml of zeocin and 100 µg/ml of spectinomycin for BP and LR transformants, respectively, followed by colony PCR in a 20 µl reaction mix with AmpliTaq DNA Polymerase (5 U/µl) (Applied Biosciences, USA) and PCR conditions as described above with primer specific annealing temperature (**Supplementary Table S1**). The recombinant plasmids were isolated using the Qiagen™ Plasmid Mini Kit (Qiagen, Germany), and the target insert sequence was verified using Sanger sequencing at Eurofins Genomics (Germany). The freeze-thaw transformation method was used to introduce the recombinant plasmid constructs into chemically competent cells of *Agrobacterium tumefaciens* strain AGL1 (Weigel and Glazebrook, 2006). The transformed bacterial colonies were selected on LA plates containing spectinomycin (100 µg/ml), carbenicillin (50 µg/ml) and rifampicin (15 µg/ml) and confirmed by colony PCR using gene-specific primers (**Supplementary Table S1**). A suspension of *A. tumefaciens* with an OD_600_ of 0.8 prepared as described by Du et al. (2014) was infiltrated into the top 3^rd^ to 5^th^ leaf of 3–4 weeks old *N. benthamiana* plants. The two *P. cactorum* genes*, Pc16451* (*PC110_g16451*) and *Pc22254* (*PC110_g22254*), previously reported to induce cell death in *N. benthamiana*, as *RXLR6* and *RXLR27*, respectively (Chen et al., 2014) were included as positive controls. In addition *INF1*, which is a known elicitor of cell death from *P. infestans* (Kamoun et al., 1997, 1998), was used as a positive control, while the empty vector pK7WG2 was used as a negative control. The expression of the recombinant *RXLR* genes in *N. benthamiana* were confirmed three days after the agroinfiltration by reverse transcription PCR (RT-PCR). Briefly, the cDNA was synthesized from the total RNA isolated from agroinfiltrated leaves and PCR was performed using 2 µl cDNA as a template, and primers targeting short fragments of the *RXLR* genes (**Supplementary Table S1**). RNA was used as a template for a negative control (-RT control).

### 2.6 Histochemical assays

Agroinfiltrated *N. benthamiana* leaves showing visible cell death were stained using trypan blue to confirm the cell death response, as trypan blue is readily taken up by dead cells but not by living cells (Keogh et al., 1980). Briefly, leaves were boiled for 5–10 minutes in the trypan blue staining solution [10 ml DL-lactic acid (90%), 10 ml phenol (equilibrated with 10 mM Tris-HCL, pH 8.0), 1 mM EDTA, 10 ml glycerol (98%), 10 ml distilled water, 20 mg trypan blue] diluted with 96% ethanol (1:1 v/v), until the green color of the leaf disappeared (Bach-Pages and Preston, 2018). The leaves were further incubated in the solution at room temperature for 30 minutes, and destained overnight in chloral hydrate solution (2.5 g/ml) on a rotary shaker (40 rpm). Additionally, 3, 3´- diaminobenzidine (DAB) and aniline blue staining (Bach-Pages and Preston, 2018) were performed to assess oxidative burst/H_2_O_2_ accumulation and callose deposition, respectively, 3 days after agroinfiltration. The insoluble brown precipitate formed after DAB staining is a result of a peroxidase dependent polymerization of DAB with H_2_O_2,_ indicating H_2_O_2_ accumulation, which is connected to cell death response (Alvarez et al., 1998; Steffens et al., 2013). Callose depositions are formed by plants upon recognition of non-self components and these fluoresce under UV light after binding with aniline blue (Jin and Mackey, 2017).

## 3 Results

### 3.1 Transcriptome profile and functional classification

From our previous defense transcriptome study of *Fragaria vesca* genotypes inoculated with *Phytophthora cactorum* (Gogoi et al., 2023a), a total of 412,970 *de novo* assembled transcript isoforms were obtained. Blasting the longest isoforms of *de novo* assembled transcripts (308,070) to the *P. cactorum* strain 10300 genome (GCA_003287315.1) resulted in 4,808 transcripts, which represent ca 1.6% of the total transcripts, of which 4,665 had more than 99% identity to *P. cactorum* genes (**Supplementary Material S1**). Three candidate RXLR effector genes, *Pc741* (*PC110_g741*), *Pc12148* (*PC110_g12148*), and *Pc19826* (*PC110_g19826*), with less than 99% nucleotide identity to the genome of *P. cactorum* strain 10300, were manually curated based on the RXLR motif and their expression during infection. These genes were included in the final list of *P. cactorum* genes, amounting to a total of 4,668 genes (**Supplementary Material S1**).

**Figure 1:**
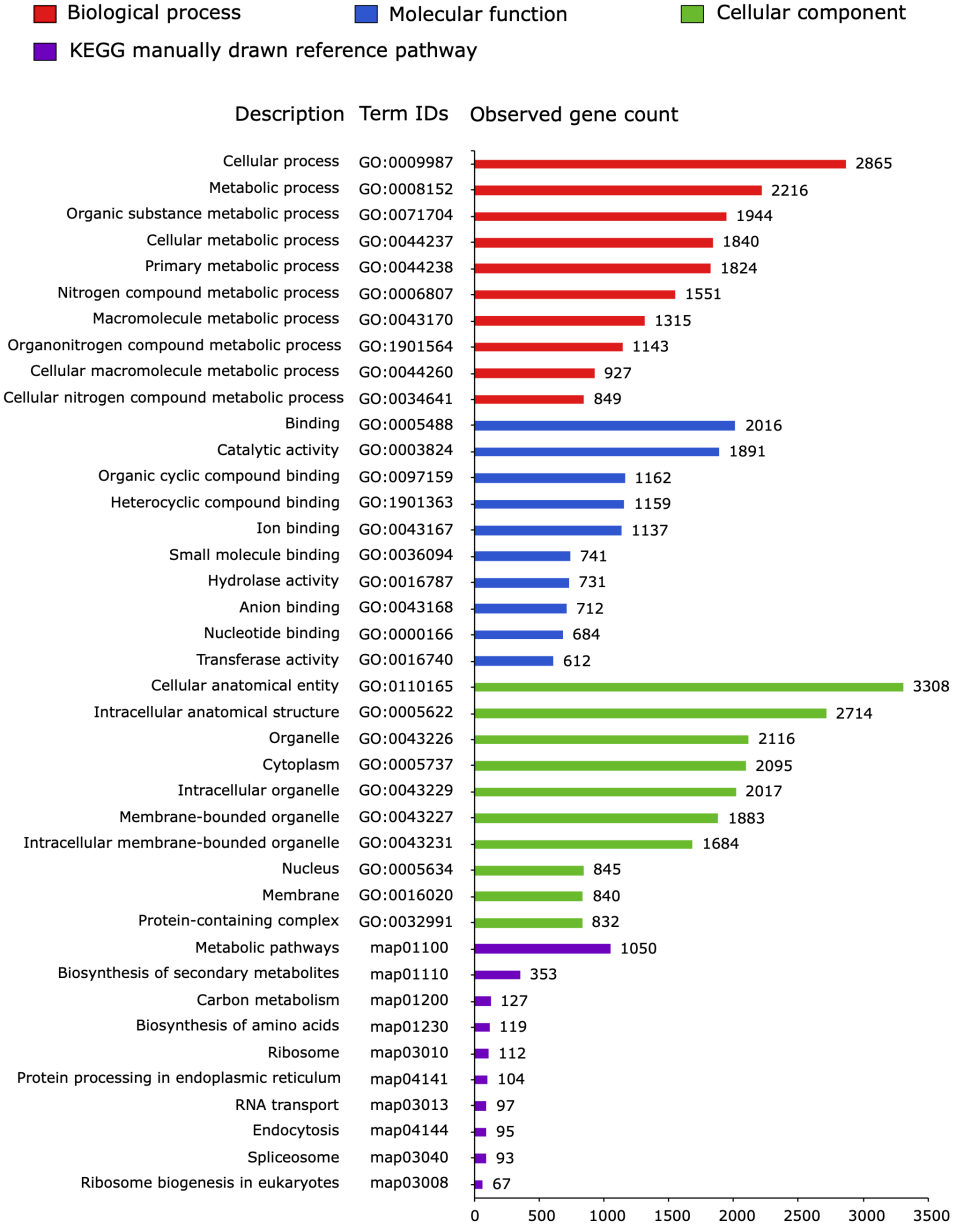
Gene ontology (GO) classification and Kyoto encyclopedia of genes and genome (KEGG) analysis of proteins encoded by transcripts of *Phytophthora cactorum* (accession GCA_003287315.1) detected in the rhizome (crown) of the susceptible *Fragaria vesca* NCGR1218, 48 hours after inoculation. The GO terms and KEGG pathways were categorized using the STRING V11.5 database (Szklarczyk et al., 2021). The top 10 most represented terms in each of the GO categories cellular component, molecular function, and biological process, as well as KEGG pathways are shown.

The 4,668 proteins encoded by the *P. cactorum* transcripts were classified using gene ontology (GO) categories, and 3,365 of them were represented in 848 ontologies (517 biological process, 188 molecular function, and 143 cellular component) while KEGG analysis identified 2,009 functional transcripts assigned to 78 pathways (**Figure 1**; **Supplementary Material S2**).

### 3.2 Secretome analysis of *P. cactorum* genes expressed during strawberry infection

Of the 4,668 proteins encoded by the detected transcripts, 539 (~12%) were predicted to have signal peptides for secretion and are thus referred to as secreted proteins (**Supplementary Material S1**). The 539 secreted proteins belonged to several effector families including 120 carbohydrate active enzymes (CAZymes), 40 RXLRs, 23 proteolytic enzymes, nine elicitins, seven cysteine rich proteins, seven necrosis inducing proteins and three crinklers (CRN) (**Table 1**; **Supplementary Table S1**). The 120 identified CAZymes belonged to 52 sub-families potentially targeting different substrates of the plant cell (**Table 2**). The majority of the sub-families belonged to glycoside hydrolases (GHs). Of the 40 RXLR effectors, 31 had complete RXLR and EER motifs while nine had only the RXLR motif (**Table 3**).

**Table 1:** Number and encoding properties of transcripts from *Phytophthora cactorum* detected 48 hours after inoculation into the crown (rhizome) of the susceptible *Fragaria vesca* NCGR1218.

Specification	Number of transcripts
*De novo* assembled transcript isoforms (strawberry and *P. cactorum*)	412,970
Longest transcript isoforms (strawberry and *P. cactorum*)	308,070
Transcripts with ≥ 72% identity to *P. cactorum* genes	4808
Transcripts with ≥ 99% identity to *P. cactorum* genes	4665
Secreted proteins^1,2^	539
Hypothetical/uncharacterized proteins	216
Carbohydrate active enzymes (CAZymes)	120
RXLR effectors	40
Proteases and peptidases (proteolytic enzymes)	23
Elicitins	9
Cysteine rich proteins	7
Necrosis inducing proteins	7
Transglutaminase elicitors	4
Crinklers (CRNs)	3
Enzyme inhibitors	3
Phytotoxin (PcF) protein	1
Others	106

^1^Secreted proteins are proteins encoded by transcripts with ≥99% identity to *P. cactorum* genes (accession GCA_003287315.1) that were predicted to be secreted (see materials and methods), and in addition include three RXLR effectors Pc741, Pc12148, and Pc19826 encoded by transcripts with 93%, 98%, and 94% identity to *P. cactorum* 10300 genes, respectively.

^2^Proteins encoded by transcripts were categorized based on the functional annotations retrieved from the dbCAN3 metaserver, effectR package, and NCBI Nr database.

**Table 2:** Carbohydrate active enzymes (CAZymes) encoded by the transcripts detected 48 hours after inoculation with *Phytophthora cactorum* in the rhizome (crown) of the susceptible *Fragaria vesca* NCGR1218.

CAZyme family	CAZyme sub-family	Substrate^1^	Gene identifier^2,3^
Auxiliary Activities (AA)	AA1_e26	Lignin	Pc19230
	AA2_e1	–	Pc2082, Pc22186
	AA3_e37	Cellulose	Pc5135, Pc14054
	AA7_e5	Chitooligosaccharide	Pc8729
	AA17_e1	Pectin	Pc19406, Pc19407, Pc19408
	AA17_e6	–	Pc16379
	AA17_e7	–	Pc5443, Pc17799
	AA17_e10	–	Pc7555, Pc7563
	AA17_e11	–	Pc2647
Carbohydrate-Binding Modules (CBM)	CBM25_e11	Starch	Pc18622
	CBM43_e4	Beta-glucan	Pc5364
Carbohydrate Esterase (CE)	CE8_e82	Pectin	Pc3935, Pc6423, Pc14408, Pc14410, Pc17084, Pc17413
Glycoside Hydrolases (GH)	GH1_e51	Beta-glucan, beta-galactan, beta-fucosides, polyphenol	Pc4696, Pc7084, Pc7987, Pc13250, Pc13251, Pc16647
	GH3_e0	Xylan, arabinan, beta-glucan, beta-glucan	Pc13195
	GH3_e87	–	Pc12010
	GH3_e146	Beta-glucan	Pc6014, Pc6015, Pc6016, Pc11468, Pc11802, Pc18158, Pc19193
	GH5_e98	–	Pc5364
	GH5_e271	–	Pc15901
	GH5_e286	Beta-mannan	Pc16655, Pc21871
	GH6_e8	–	Pc19276, Pc19280
	GH7_e0	Cellulose, chitosan, cellulose	Pc4344, Pc7120
	GH10_e40	Xylan	Pc16323
	GH12_e27	Xyloglucan	Pc4383, Pc4387, Pc4389, Pc4391, Pc11987
	GH12_e34	Cellulose, xyloglucan	Pc6604, Pc19081
	GH16_e194, GH16_e295	–	Pc1823, Pc9318, Pc9321, Pc9332, Pc9333, Pc17088
	GH17_e49	–	Pc5033
	GH17_e70	–	Pc13477, Pc13479, Pc17453, Pc19481
	GH28_e98	Pectin	Pc12321, Pc13557, Pc13558, Pc13560, Pc13562, Pc20086, Pc20087
	GH30_e22	Arabinogalactan protein	Pc3032
	GH30_e27	Beta-glucan	Pc1071, Pc1076, Pc1077, Pc2660, Pc8481
	GH31_e39	Starch	Pc3876
	GH31_e73	Starch, xyloglucan	Pc18622
	GH32_e120	Fructan	Pc9952, Pc9954
	GH38_e30	Host glycan	Pc33
	GH43_e141	Arabinan	Pc13374
	GH53_e5	Arabinogalactan protein	Pc3669, Pc3670, Pc3671
	GH54_e0	Arabinan, xylan	Pc4440
	GH72_e7	–	Pc11788
	GH72_e8	–	Pc16362, Pc18959
	GH78_e20	–	Pc5615
	GH78_e52	Pectin	Pc6551
	GH81_e9	–	Pc5328
	GH105_e34	–	Pc9937
	GH131_e1	–	Pc1747, Pc11593, Pc12878
	GH140_e15	–	Pc12261
GlycosylTransferases (GT)	GT24_e1	–	Pc2136
	GT31_e29	–	Pc20475
	GT60_e0	–	Pc12475, Pc20397
	GT71_e30	–	Pc20024
Polysaccharide Lyases (PL)	PL1_e43	Pectin	Pc6790, Pc13372, Pc13385, Pc16314, Pc18155
	PL3_e16	Pectin	Pc12532, Pc14174, Pc14175, Pc15713, Pc16232, Pc16949, Pc18074, Pc18639, Pc21171
	PL4_e12	Pectin	Pc14972, Pc19106, Pc19107, Pc19109

^1^ - indicate no available information about enzyme substrate in the dbCAN3 metaserver.

^2^Full length sequences of the proteins encoded by detected transcripts were retrieved using NCBI protein sequences (GCA_003287315.1_Pcac_10300_v1_protein), and these were used for CAZyme analysis in the dbCAN3 metaserver.

^3^Pc is the abbreviated form of PC110_g in the gene identifier and was obtained from *Phytophthora cactorum* accession GCA_003287315.1.

**Table 3:** RXLR effector candidates encoded by transcripts detected 48 hours after inoculation with *Phytophthora cactorum* in the rhizome (crown) of the susceptible *Fragaria vesca* NCGR1218.

RXLR gene ID^1,2^	Mean expression value at 48 hpi^4^	Protein length (amino acid)	RXLR start position	RXLR…. EER motif	Induce cell death^5^
Pc741^3^	1.7	390	46	**RFLR**ATBAAD**EER**	Yes
Pc1530	0.4	120	42	**RHLR….**	–
Pc2488	0.4	214	43	**RSLR**TAETNG**EER**	–
Pc3969	0.7	199	45	**RFLR….**	–
Pc4180	0.8	116	109	**RRLR….**	–
Pc5187	1.1	139	116	**RFLR….**	–
Pc6139	0.3	1538	49	**RNLR**ATATTNG**EER**	–
Pc7604^3^	0.9	146	48	**RYLR**SRKTIDGDTQA**EER**	No
Pc7628^3^	1.8	171	54	**RFLR**GESKIQNLTGGDRDEA**EER**	No
Pc8318^3^	2.2	371	53	**RSLR**RYED**EER**	Yes
Pc8682^3^	4.7	162	30	**RRLR….**	No
Pc9279	0.5	161	46	**RFLR**TQKAIEKYDEEE**EER**	–
Pc10890^3^	18.3	147	48	**RFLR**SHQTTGDEGKITEHDD**EER**	Yes
Pc11254^3^	1.4	349	42	**RSLR**IGYITKEDD**EER**	No
Pc12148	0.3	132	53	**RFLR**NQEDEEDLDEEDEEDEEDEED**EER**	–
Pc12728^3^	0.3	290	56	**RFLR**NHDD**EER**	No
Pc15067^3^	1.4	159	58	**RFLR**GNAIKDLTTADNDSDAKD**EER**	No
Pc16443	0.3	104	45	**RSLR….**	–
Pc16451^3^	1.7	260	50	**RFLR**SKHHEQDNVKDAEG**EER**	Yes
Pc16706^3^	0.7	202	41	**RLLR**TATMSDD**EER**	No
Pc16877^3^	1.2	136	56	**RLLR**ADGAGDDKLPAEE**EER**	No
Pc17244^3^	2.4	132	41	**RSLR**SHTDR**EER**	No
Pc17901	0.4	233	42	**RALR**TYTEASKDG**EER**	–
Pc18286	0.8	169	55	**RYLR….**	–
Pc18769	0.6	407	52	**RFLR**TYTTERAVSN**EER**	–
Pc19202^3^	1.9	184	49	**RRLR**KHDSKVDLESDD**EER**	No
Pc19237	0.9	134	48	**RFLR**KESVKNNEAID**EER**	–
Pc19826	0.4	292	55	**RFLR**ATAQTYDGDDNS**EER**	–
Pc19898	1.4	195	43	**RHLR**AEIRIDYDNNNASD**EER**	–
Pc19924	0.5	244	55	**RFLR**IETTIEEEDSEDD**EER**	–
Pc20579	0.3	276	51	**RSLR**AEKVIEVGNEN**EER**	–
Pc20589^3^	26.7	132	43	**RLLR**SYSKPVEDDSDDLDDS**EER**	No
Pc20813^3^	28.4	198	57	**RLLR**SEFVPADDAVDDE**EER**	Yes
Pc21899^3^	44.9	142	50	**RSLR**YHGNDDRADEEEDEED**EER**	No
Pc22014^3^	33.5	145	54	**RFLR**TNDEEDAPEEDDEDFS**EER**	No
Pc22183	1.1	54	30	**RSLR….**	–
Pc22254^3^	19.6	133	52	**RFLR**VTGPEDAD**EER**	Yes
Pc22290^3^	2.1	177	52	**RLLR….**	Yes
Pc22490	0.5	109	44	**RFLR**SVKTEDDG**EER**	–
Pc22506	0.8	222	50	**RLLR**RYDDD**EER**	–

^1^Full length sequences of the proteins encoded by detected transcripts were retrieved using NCBI protein sequences (GCA_003287315.1_Pcac_10300_v1_protein), and these were used to identify RXLR effectors using the effectR program.

^2^Pc is the abbreviated form of PC110_g in the RXLR ID and was obtained from *Phytophthora cactorum* accession GCA_003287315.1.

^3^RXLR effectors selected for cloning and transient expression in *Nicotiana benthamiana*.

^4^hpi hours post inoculation.

^5^Cell death response induced upon transient expression of RXLR effectors in *N. benthamiana* at five days post agroinfiltration; ‘-’ indicates the RXLR effectors that were not tested.

**Figure 2:**
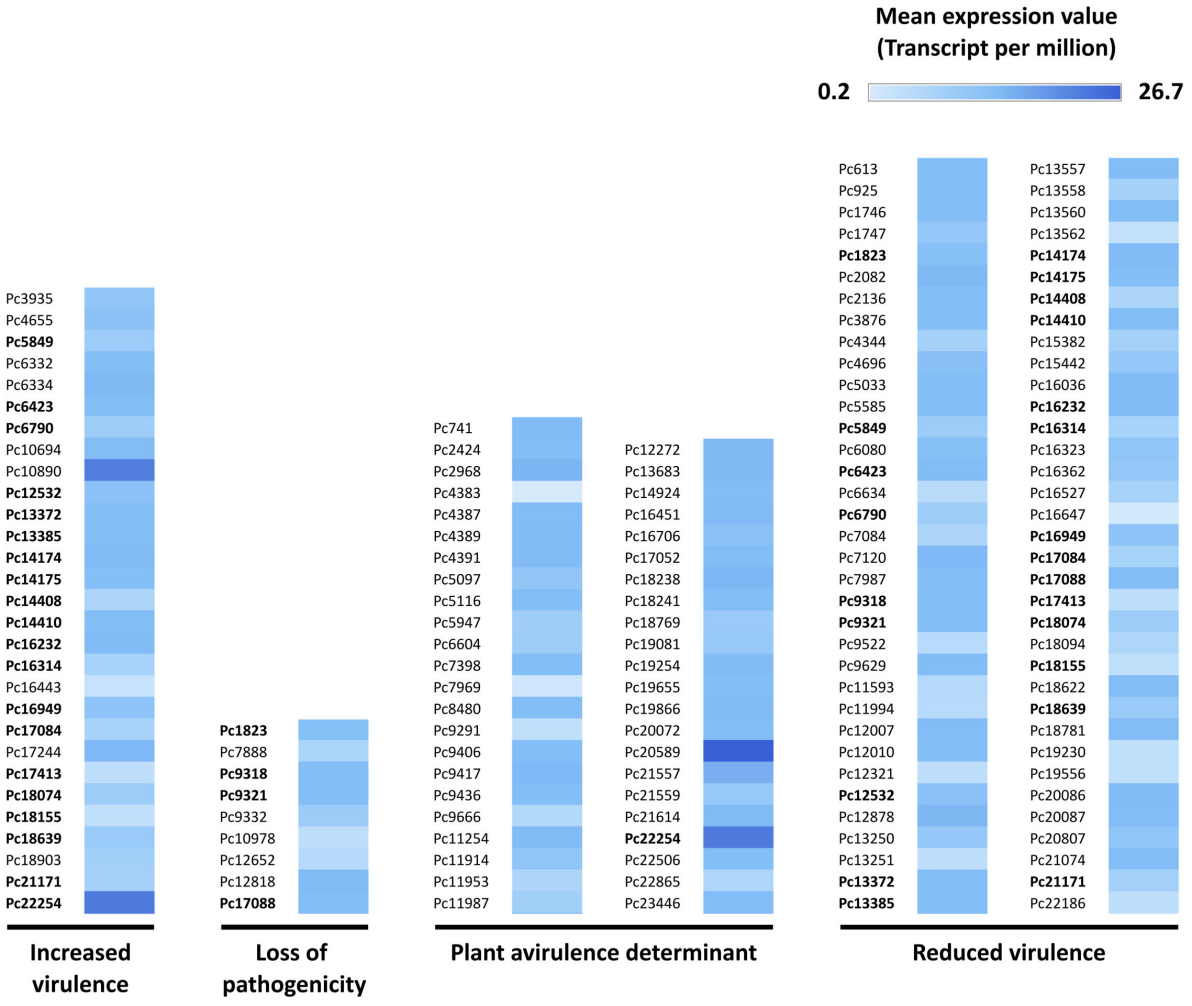
Secreted *Phytophthora cactorum* proteins with similarity to proteins that influence pathogen host interaction (PHI), based on the PHI-base (Urban et al., 2022). The proteins are deduced from transcripts detected in the rhizome (crown) of a susceptible *Fragaria vesca* genotype (NCGR1218), 48 hours after inoculation with *P. cactorum*. The PHI-phenotypes are resulting from a mutation or altered expression of the specific gene in the pathogen while ‘plant avirulence determinant’ represents an effector required for recognition of a pathogen by the resistant host. Pc is the abbreviated form of PC110_g in the gene identifier and is obtained from *P. cactorum* accession GCA_003287315.1. The gene IDs in bold are assigned to more than one PHI-phenotype.

*In silico* analysis of the secreted proteins of *P. cactorum* using the pathogen host interaction database (PHI-base) showed 129 proteins having 30–99% sequence identity with proteins known to influence host-pathogen interaction in oomycete, fungal, bacterial, or nematode species. These 129 proteins were categorized in different PHI-phenotypes, of which 70 were assigned to the ‘reduced virulence’ phenotype, 45 to ‘plant avirulence determinant’, 29 to ‘increased virulence’, and nine to ‘loss of pathogenicity’ (**Figure 2**) (**Supplementary Material S3**). Twenty-four of the proteins were assigned to more than one PHI-phenotype.

### 3.3 RXLR effectors that trigger cell death in *Nicotiana benthamiana*

**Figure 3:**
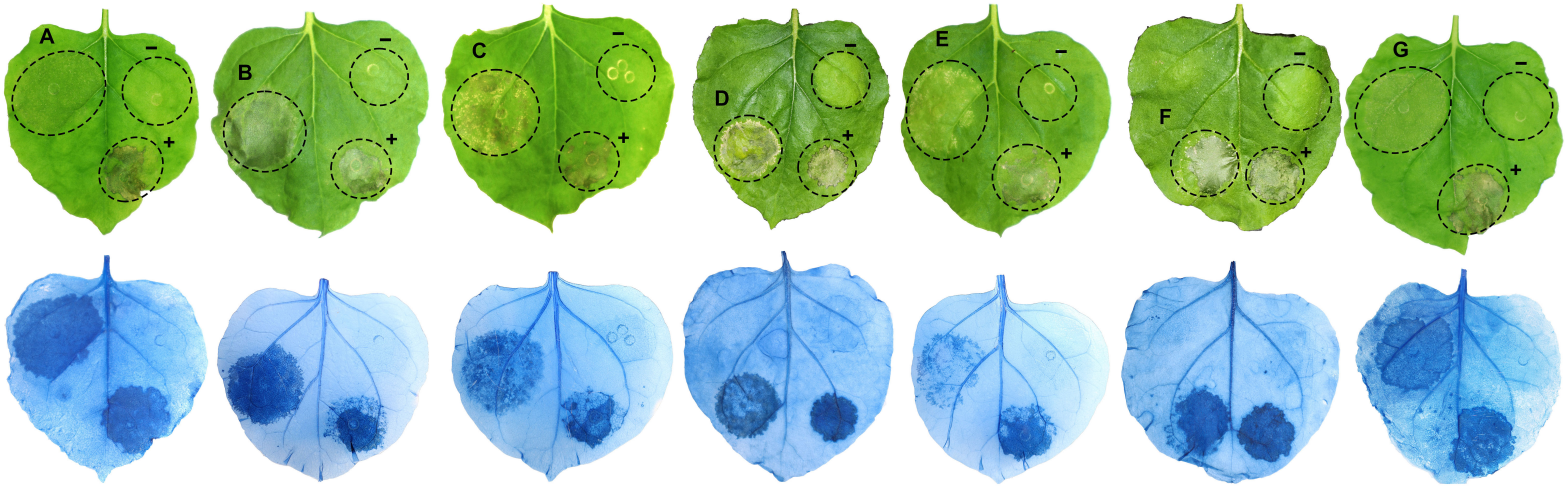
Cell death induced by *Phytophthora cactorum* RXLR effector genes **(A)** *Pc741*, **(B)** *Pc8318*, **(C)** *Pc10890*, **(D)** *Pc16451*, **(E)** *Pc20813*, **(F)** *Pc22254*, and **(G)** *Pc22290* after transient expression in *Nicotiana benthamiana* leaves. The lower panel confirms cell death response after trypan blue staining. *INF1*, an elicitor gene of cell death from *Phytophthora infestans* was used as a positive control (+), and the empty vector pK7WG2 was used as a negative control (-) in each of the leaves. In addition, two previously reported cell death inducing RXLR genes **(D)** *Pc16451* and **(F)** *Pc22254* from *P. cactorum* (Chen et al., 2014) were included as positive controls. Images were taken five days after agroinfiltration of the gene constructs and after trypan blue staining of the same leaf. Pc is the abbreviated form of PC110_g from the gene identifier and is obtained from *P. cactorum* accession GCA_003287315.1.

Twenty of the 40 candidate *RXLR* effector genes with high expression values during infection of strawberry or with high similarity to known *Phytophthora* effectors were selected for transient expression in *N. benthamiana* (**Table 3**). The expression of the agroinfiltrated *RXLR* effector genes in *N. benthamiana* were confirmed three days post agroinfiltration using RT-PCR (**Supplementary Figure S1**), while the phenotypic responses of the expressed *RXLR* genes were recorded five days post agroinfiltration. Five previously unreported *RXLR* effector genes, *Pc741* (*PC110_g741*), *Pc8318* (*PC110_g8318*), *Pc10890* (*PC110_g10890*), *Pc20813* (*PC110_g20813*), and *Pc22290* (*PC110_g22290*) induced cell death when expressed in *N. benthamiana* leaves, which was confirmed with trypan blue staining (**Figure 3**). The cell death induced by the five *RXLR* effectors were further confirmed by 3, 3’- diaminobenzidine (DAB) staining, which showed a strong brown color precipitation in the *RXLR* agroinfiltrated regions, indicating an accumulation of H_2_O_2_ compared to the regions agroinfiltrated with the empty vector control (**Supplementary Figure S2**). Furthermore, aniline blue staining showed fluorescence in the *RXLR* agroinfiltrated regions with induced cell death, demonstrating accumulation of callose, in contrast to the regions agroinfiltrated with empty vector control (**Supplementary Figure S3**). These results indicate that the five RXLR effectors could activate the immune system of *N. benthamiana*.

The protein template modeling (TM)-score matrix for RXLR effectors revealed that the TM-scores for 99.6% of the pairwise comparisons of effectors were 0.5 or below (**Supplementary Material S4**), suggesting that they have different structures. For the five RXLRs that induced cell death in *N. benthamiana* (Pc741, Pc8318, Pc10890, Pc20813, and Pc22290), the TM scores were less than 0.4 in pairwise comparison with each other. Three RXLR effector pairs gave TM scores slightly above 0.5, which could indicate some structural similarity. However, the scores were all below 0.6, so not very confident. Phylogenetic analysis of these five RXLRs with homologs from 23 different *P. cactorum* strains revealed that most of the homologs from strawberry strains were highly similar. Some of the homologs of Pc741, Pc8318, and Pc22290 from the apple strains were phylogenetically distinct from particularly the crown rot strains (**Supplementary Figure S4**). Homologs of Pc10890 were detected only in 16 of the *P. cactorum* genomes available, and these were all identical (**Supplementary Material S5**).

In the cell death inducing RXLR effectors, from zero to four sites under positive selection were detected and from zero to six sites under negative selection (**Supplementary Material S6**). The RXLR effector Pc20813, which was among the *P. cactorum* genes with highest expression (13 to 17 fold higher than Pc741, Pc8318 and 22290), had exclusively positive selection sites compared to its homologs.

## 4 Discussion

In this study, the transcriptome of the strain *P. cactorum* 10300 was investigated during infection of the susceptible *Fragaria vesca* genotype NCGR1218 at 48 hours post inoculation, which is an early and presumably important phase of the infection (Toljamo et al., 2016; Gogoi et al., 2023a). No visible necrotic lesions were observed at this time point, which is consistent with the previous report on the Hawaii4 genotype, where only hyphal growth was visible on the root surfaces after inoculation with *P. cactorum* (Toljamo et al., 2016). Our study focused on the transcriptome profile of *P. cactorum* during infection of its natural host, while previous transcriptomics studies were either based on different life stages of *P. cactorum*, infection of model hosts like *Nicotiana benthamiana, N. tabacum*, and *Solanum lycopersicum*, or strawberry *in vitro* (Chen et al., 2014, 2018; Nellist et al., 2021). In total 4668 *P. cactorum* transcripts encoding proteins were detected. GO and KEGG analysis indicated that most of the proteins encoded by the transcripts had binding and catalytic activity and were involved in the metabolism of the pathogen (**Figure 1**), but 539 (˜12%) were predicted to encode secreted effector proteins belonging to different apoplastic and cytoplasmic effector groups (**Table 1**; **Supplementary Material S1**). Based on analysis of the secreted proteins encoded by the detected *P. cactorum* transcripts in the pathogen-host interaction (PHI)-database (Urban et al., 2022), 129 of 539 secreted proteins were 30–99% identical to proteins previously demonstrated to have influence on virulence of different pathogens, including *Phytophthora* spp. (**Figure 2** **;** **Supplementary Material S3**).

As much as 40% of the predicted secreted proteins encoded by *P. cactorum* transcripts detected during strawberry infection were hypothetical proteins with unknown function. Of the predicted secreted proteins with a putative function, most belonged to typical apoplastic effector groups of which the largest was carbohydrate active enzymes (CAZymes) that are known to participate in host cell wall degradation and metabolism to facilitate infection (Ospina-Giraldo et al., 2010). Of the 120 proteins belonging to the CAZymes, the majority were glycoside hydrolases (GHs). The GHs secreted by plant associated fungi and oomycetes represent the largest class of CAZymes (Bradley et al., 2022). Numerous CAZymes belonging to different families including AA17, CBM25, GH3, GH5, GH6, GH7, GH10, GH12, GH16, GH17, GH28 detected in this study have previously been described as virulence factors in different pathogens (Hardham and Blackman, 2018; Rafiei et al., 2021; Sabbadin et al., 2021; Bradley et al., 2022). For instance, the xylanases PpXYn1 and PpXyn2, belonging to the GH10 family, are virulence factors that degrade xylan and are upregulated during *Phytophthora parasitica* infection (Lai and Liou, 2018). However, some of the CAZymes that act as virulence factors may also elicit defense responses in the host. For example, the cellobiohydrolase PsGH7 and the xyloglucanase PsXEG1 (GH12 family) from *Phytophthora sojae* are virulence factors that promote infection, while they also elicit hypersensitive response in the soybean host, due to recognition as pathogen-associated molecular patterns (PAMPs) (Ma et al., 2015; Tan et al., 2020).

In addition to CAZymes, twenty-three proteolytic enzymes including proteases and peptidases were identified in our study. Proteolytic enzymes are important virulence factors in plant-pathogen interactions and may disrupt the host defense for successful invasion (Jashni et al., 2015). For instance, the cysteine protease genes *PpCys44* and *PpCys45* of *P. parasitica* have been shown to act as virulence factors during infection in *N. benthamiana* (Zhang et al., 2020). Three enzyme inhibitors, Kazal-type serine protease inhibitor, protease inhibitor epic4 and elastase-like inhibitors, were also identified. This group of proteins, particularly the protease inhibitors are known to bind and inhibit the function of host apoplastic proteases during plant colonization (Jashni et al., 2015). For instance, an extracellular kazal-like serine protease inhibitor EP1 from *P. infestans* has been shown to inhibit and interact with the pathogenesis-related P69B subtilisin-like serine protease of tomato (Tian et al., 2004). Similarly, protein inhibitors like *EPIC* genes were shown to be upregulated during plant infection and their products are known to inhibit papain-like cysteine proteases (Tian et al., 2007; Kaschani et al., 2010).

Furthermore, nine elicitins, seven cysteine rich proteins, seven necrosis inducing proteins (NPPs), four transglutaminase elicitors, and one phytotoxin protein were identified. Cysteine rich proteins, elicitins, and NPPs in general are considered PAMPs that may trigger immunity in host plants leading to cell death (Orsomando et al., 2001; Naveed et al., 2020; Midgley et al., 2022). However, some of these proteins also function as virulence factors in different pathogens. For instance, cysteine rich protein (SCR96) in *P. cactorum* (Chen et al., 2016b), elicitin protein (β-cinnamomin) in *P. cinnamomi* (Horta et al., 2010; Islam et al., 2019), cell wall transglutaminase elicitor in *P. infestans* (Brus-Szkalej et al., 2021), and phytotoxic protein SCR82 in *P. capsici* (Zhang et al., 2021) function as pathogen virulence factors in addition to triggering cell death as a result of PAMP recognition.

Of cytoplasmic effector groups, only three CRNs were identified in our study, of which PC110_g7969 showed 81% identity (86% similarity) to CRN1 from *P. infestans* that trigger necrotic responses in *N. benthamiana* and the host plant tomato (Torto et al., 2003). CRNs are known to result in cell death and chlorosis when expressed in the plant, however some of the CRNs can also suppress cell death, thereby promoting virulence. For instance, *P. parasitica* CRN effector PpCRN7 enhances INF1 induced cell death in *N. benthamiana*, while PpCRN20 suppresses it. Despite their contrasting functions, both PpCRN7 and PpCRN20 increase plant susceptibility to *P. parasitica* (Maximo et al., 2019).

Forty RXLR candidates were detected in the transcripts, which constitutes only 20% of the predicted RXLRs in the genome of *P. cactorum* (Armitage et al., 2018). This was 10% less than detected by Nellist et al. (2021) at 48h after inoculation, but they used tissue culture plants which are much more fragile than the plants used in our study thus promoting a more rapid infection process. Twenty of the 40 detected RXLR candidates were transiently expressed in *N. benthamiana* to investigate their cell death inducing properties and five previously unreported RXLR effector genes, *Pc741, Pc8318, Pc10890, Pc20813*, and *Pc22290*, induced cell death. Interestingly, *Pc22290*, an RXLR candidate without the EER motif induced cell death. The EER motif supposedly helps in translocation of RXLR effectors into the host cell, but an exact RXLR-EER sequence is not a requirement for its translocation (Dou et al., 2008; Van West et al., 2010; Chen et al., 2020). RXLR effectors that lack the EER motif have also previously been shown to induce cell death, e.g., the avirulence protein ATR13 from the oomycete pathogen *Hyaloperonospora parasitica* induced cell death in *Arabidopsis* (Allen et al., 2004).

The cell death region induced by RXLR effectors in *N. benthamiana* leaves showed accumulation of reactive oxygen species (ROS) such as H_2_O_2_ and callose deposits as confirmed by 3, 3’- diaminobenzidine (DAB) and aniline blue staining, respectively. Similar responses have been observed for RXLR effectors from other *Phytophthora* species (Yin et al., 2019; Situ et al., 2020). ROS like H_2_O_2_ strengthen the plant cell walls through oxidative cross-linking and also act as signaling molecules that induce defense responses (Alvarez et al., 1998; Steffens et al., 2013; Redza-Dutordoir and Averill-Bates, 2016). Furthermore, callose deposition is one of the immune responses deployed by plants upon recognition of ‘non-self’ components and is induced during PTI (Du et al., 2015). It is not yet clear whether the cell death induced is the direct effect of the RXLR effectors or due to recognition of the effector by resistance proteins in *N. benthamiana.* However, deposition of callose in the RXLR expressing leaves suggests that cell death is a result of host defense response (Jin and Mackey, 2017). It should be noted that these RXLR effectors not necessarily induce similar effects in strawberry, but the response in *N. benthamiana* is an indication of their relevance.

Since the RXLR effectors are known to target a wide range of cellular processes, the protein sequences of the identified cell death inducing RXLR effectors from *P. cactorum* were blasted against the UniProt database to look for similarities with functionally studied proteins from other pathogens. The RXLR effector Pc741 showed 44% sequence identity (54% similarity) to the *P. capsici* RXLR207 that induced ROS-mediated cell death in transgenic *Arabidopsis* and reduced pathogen colonization. However, a mutation in RXLR207 resulted in decreased pathogen virulence, suggesting it is crucial for infection (Li et al., 2019). The Pc8318 RXLR effector showed 54% identity (68% similarity) to the RXLR effector CRE5 (PITG_06308) of *P. infestans*, which did not induce cell death but in contrast suppressed cell death induced by Avh238, Avh241, BAX, INF1, thus indicating its potential roles in the virulence of *Phytophthora* spp (Yin et al., 2017). The Pc10890 RXLR effector showed 66% identity (77% similarity) with RXLR effector SFI6 from *P. infestans*, which like CRE5 did not induce cell death, but suppressed PAMP-triggered immunity in *Arabidopsis* and tomato, and increased virulence of *P. infestans* in *N. benthamiana* (Zheng et al., 2014). The Pc20813 RXLR effector showed 31% identity (47% similarity) with *P. infestans* RXLR effector SFI2 that induces cell death in *N. benthamiana* but attenuates flg22-induced immune response in tomato and Arabidopsis (Zheng et al., 2014). Furthermore, the Pc22290 RXLR effector showed 36% identity (53% similarity) with *P. parasitica* effector RXLR3 that did not induce cell death but significantly increased *Phytophthora* infection, and moderately suppressed INF1 induced cell death in *N. benthamiana* (Dalio et al., 2018). Although the five RXLR effectors induced cell death in *N. benthamiana*, it has yet to be confirmed if the induced cell death can inhibit *P. cactorum* infection. Thirteen of the 20 cloned RXLR effectors did not induce cell death in *N. benthamiana* indicating that these RXLRs do not directly activate the plant’s immune system. Some RXLR effectors that do not trigger cell death, instead can suppress PTI or ETI induced cell death thereby promoting pathogen virulence (Wang et al., 2023). The cell death suppression effect of RXLRs can be studied by co-infiltration with known effectors or elicitins that induce cell death in *N. benthamiana* (Zhan et al., 2022).

Two of the RXLR effectors that induced cell death in *N. benthamiana*, Pc741 and Pc22254, were assigned to the PHI-phenotype ‘plant avirulence determinant’, hence confirming the prediction from the PHI database and demonstrating that it can be a useful tool for selection of effector candidates for further characterization and functional studies.

Only a few *P. cactorum* effectors have been functionally characterized so far: four RXLRs, one NPP, two elicitins, and four cysteine rich proteins (Chen et al., 2023). The five additional cell death inducing RXLRs identified in this study can in principle play important roles in pathogenicity, despite being recognized by *N. benthamiana* when transiently expressed. However, the direct roles of the RXLR effectors identified in this study, in the virulence of *P. cactorum* remain to be elucidated. The protein structural similarity analysis of the identified RXLR effectors indicated that 99.6% of them were structurally unrelated with TM scores 0.5 or below. This included the RXLRs that induced cell death in *N. benthamiana*. Homology analysis revealed that all cell death inducing effectors were conserved across different strains of *P. cactorum*, but that some of the homologs of Pc741, Pc8318, and Pc22290 from the apple strains were phylogenetically distinct from particularly the crown rot strains (**Supplementary Figure S4** **,** **Supplementary Material S5**). The RXLR effector Pc20813 exhibited only sites under positive selection, indicating that these amino acid residues have an important role in the host-pathogen interaction (Sironi et al., 2015). The *Pc20813* gene was among the genes with highest expression during the infection and displayed greater divergence among its homologs in *P. cactorum* strains than the other cell death inducing effectors. This contrasts with previous findings that suggest genes with higher expression levels tend to diverge less than those with lower expression (Pál et al., 2001; Yang et al., 2012; Williamson et al., 2014). In three other RXLRs (Pc741, Pc8318 and Pc22290), more negative selection sites were observed suggesting that these genes might be under strong negative selection favoring amino acid substitutions that can affect pathogen’s fitness.

Previous studies have reported that pathogens drive effector evolution through mutation and sequence substitution in *RXLR* effector genes, thus promoting their virulence (Win and Kamoun, 2007; Jiang et al., 2008; Dong et al., 2009; Qutob et al., 2009; Cui et al., 2012). Further analysis of polymorphisms in the equivalent RXLR effector genes from other strains of *P. cactorum* could therefore provide more insights about their important domains and evolution of virulence mechanisms. In addition, identification of the host receptors or protein interacting partners of the identified cell death inducing RXLR effectors could help to understand signaling pathways involved in cell death and provide insights into resistance mechanism involved. These insights can further help in screening of resistant strawberry genotypes and development of future disease control strategies.

## 5 Conclusion

The present transcriptome study provides comprehensive insights of the *Phytophthora cactorum* genes expressed during the early stage of infection of the rhizome of the model plant, *Fragaria vesca*. A total of 4668 *P. cactorum* transcripts were identified, and 539 of these were predicted to encode secreted proteins belonging to different effector families including CAZymes, elicitins, cysteine rich proteins, necrosis inducing proteins, proteolytic enzymes and RXLRs. Twenty of the 40 RXLR effectors identified were transiently expressed in *Nicotiana benthamiana*, and five previously unreported RXLR effectors triggered cell death response. The functional roles of these RXLR effectors in strawberry infection is not yet known and need further investigation. Further research on the subcellular localization of these RXLR proteins in the plant cell, and interactions of host proteins and the RXLR effectors of *P. cactorum* can help to develop new strategies for breeding resistance in strawberry.

## Data availability statement

The data presented in the study are deposited in the ArrayExpress repository, accession no. E-MTAB-12152, released on 1 January 2023. https://www.ebi.ac.uk/biostudies/arrayexpress/studies/E-MTAB-12152.

## Author contributions

BG: Methodology, Formal Analysis, Writing – original draft, Investigation. AG: Writing – original draft, Methodology, Investigation, Formal Analysis. MP: Writing – review & editing, Investigation. AS: Writing – review & editing. MB: Writing – original draft, Supervision, Project administration, Investigation, Funding acquisition, Conceptualization.
