# Supplementary material for: Transcriptome analysis of Phytophthora cactorum infecting strawberry identified RXLR effectors that induce cell death when transiently expressed in Nicotiana benthamiana
Source: Front Plant Sci. 2024 May 24;15:1379970. doi: 10.3389/fpls.2024.1379970 (PMC11157022; doi:10.3389/fpls.2024.1379970)
Supplement: Supplementary file 1 [file DataSheet_1.docx]

Supplementary Material

# Supplementary Data

**Supplementary Material S1** – excel sheet with transcriptome data including annotations

**Supplementary Material S2** – excel sheet with mapping, and Gene ontology (GO) and Kyoto encyclopedia of genes and genome (KEGG) analysis

**Supplementary Material S3** – excel sheet with PHI-phenotype analysis

**Supplementary Material S4** – excel sheet with Template modeling score matrix of RXLR effectors

**Supplementary Material S5** – excel sheet with identity matrixes of homologs of cell death inducing RXLR effectors

**Supplementary Material S6** – excel sheet with substitution rates for cell death inducing RXLR effectors

# Supplementary Figures and Tables

## Supplementary Figures


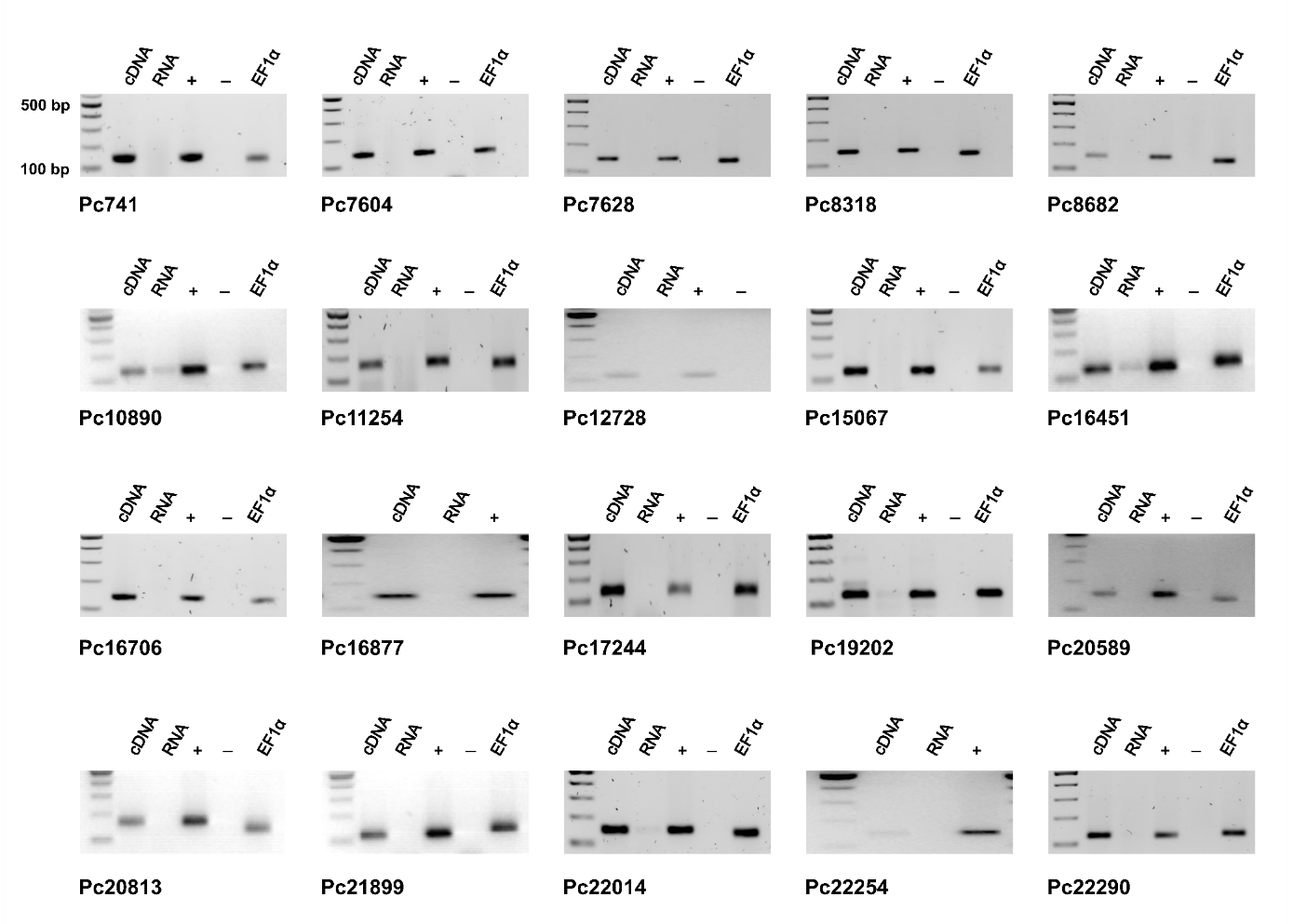


**Supplementary Figure S1.** Expression of *Phytophthora cactorum* RXLR effector genes in agroinfiltrated leaves of *Nicotiana benthamiana,* confirmed by reverse transcription PCR (RT-PCR). Each of the twenty gel images shown here represent RT-PCR products amplified using gene-specific primers for the cloned *RXLR* genes and the internal control gene *Elongation factor 1 alpha* (*EF1α*) of *N. benthamiana*. In each of the gels, the 100 bp ladder (New England BioLabs) was used as a size marker. cDNA was used as a PCR template to confirm gene expression, while RNA was used as a control template to detect possible plasmid DNA contamination in the RNA (=minus reverse transcriptase control; lanes marked RNA). Recombinant plasmid DNA harbouring each *RXLR* gene in pK7WG2 was used as a positive control (+) for PCR and water was used as a minus template control (-). Pc is the abbreviated form of PC110_g in the gene identifier and is obtained from *P. cactorum* Accession GCA_003287315.1.

*
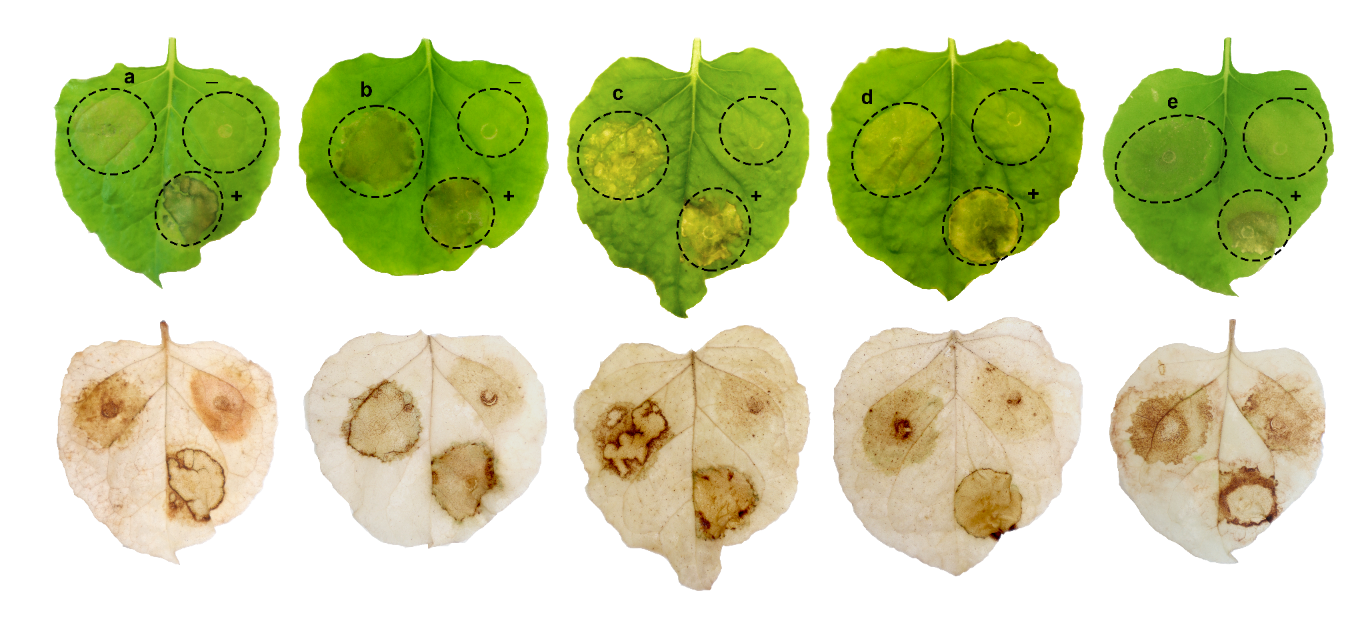
***Supplementary Figure S2.** Accumulation of reactive oxygen species (H_2_O_2_) induced by *Phytophthora cactorum* RXLR effectors genes (a) *Pc741,* (b) *Pc8318,* (c) *Pc10890,* (d) *Pc20813,* and (e) *Pc22290* after transient expression in *Nicotiana benthamiana* leaves. The brown precipitate in the cell death region confirms accumulation of H_2_O_2_ after 3, 3’- diaminobenzidine (DAB) staining (lower panel). *INF1*, an elicitor gene from *Phytophthora infestans* was used as a positive control (+) for cell death, while the empty vector pK7WG2 was used as a negative control (-) in each of the leaves. Images were taken five days after agroinfiltration of the constructs and after DAB staining of the same leaf. Pc is the abbreviated form of PC110_g from the gene identifier obtained from *P. cactorum* accession GCA_003287315.1.


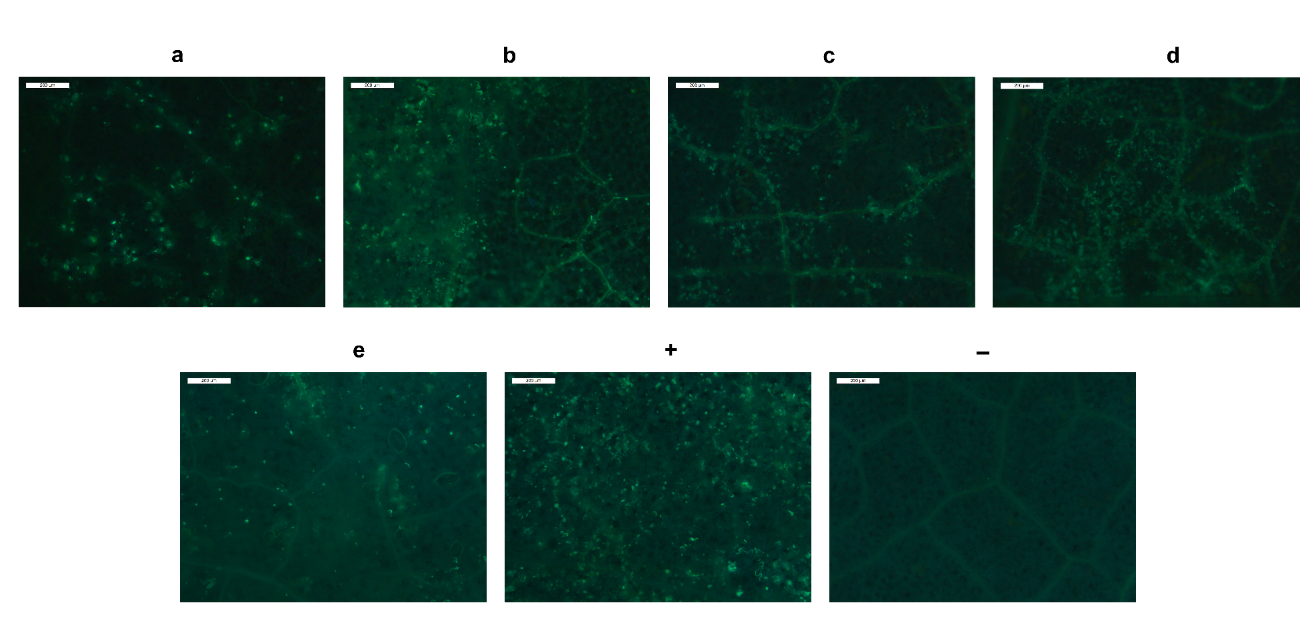


**Supplementary Figure S3.** Callose deposition induced by *Phytophthora cactorum* RXLR effector genes (a) *Pc741*, (b) *Pc8318*, (c) *Pc10890*, (d) *Pc20813*, and (e) *Pc22290* after transient expression in *Nicotiana benthamiana* leaves. Fluorescence spots confirms callose depositions after aniline blue staining. *INF1*, an elicitor gene from *Phytophthora infestans* was used as a positive control (+) for cell death, and the empty vector pK7WG2 was used as a negative control (-). Samples were harvested five days after agroinfiltration of the recombinant constructs. Images were taken using Fluorescence Microscope DFC425 (Leica Microsystems, Germany) with an I2 filter cube (450-490 bandpass excitation filter, 510 nm dichromatic mirror, and 515 nm suppression filter) and exposure time 410 ms viewed under 5x objective lens (scale bar 200 µm). Pc is the abbreviated form of PC110_g from the gene identifier obtained from *P. cactorum* accession GCA_003287315.1.

##
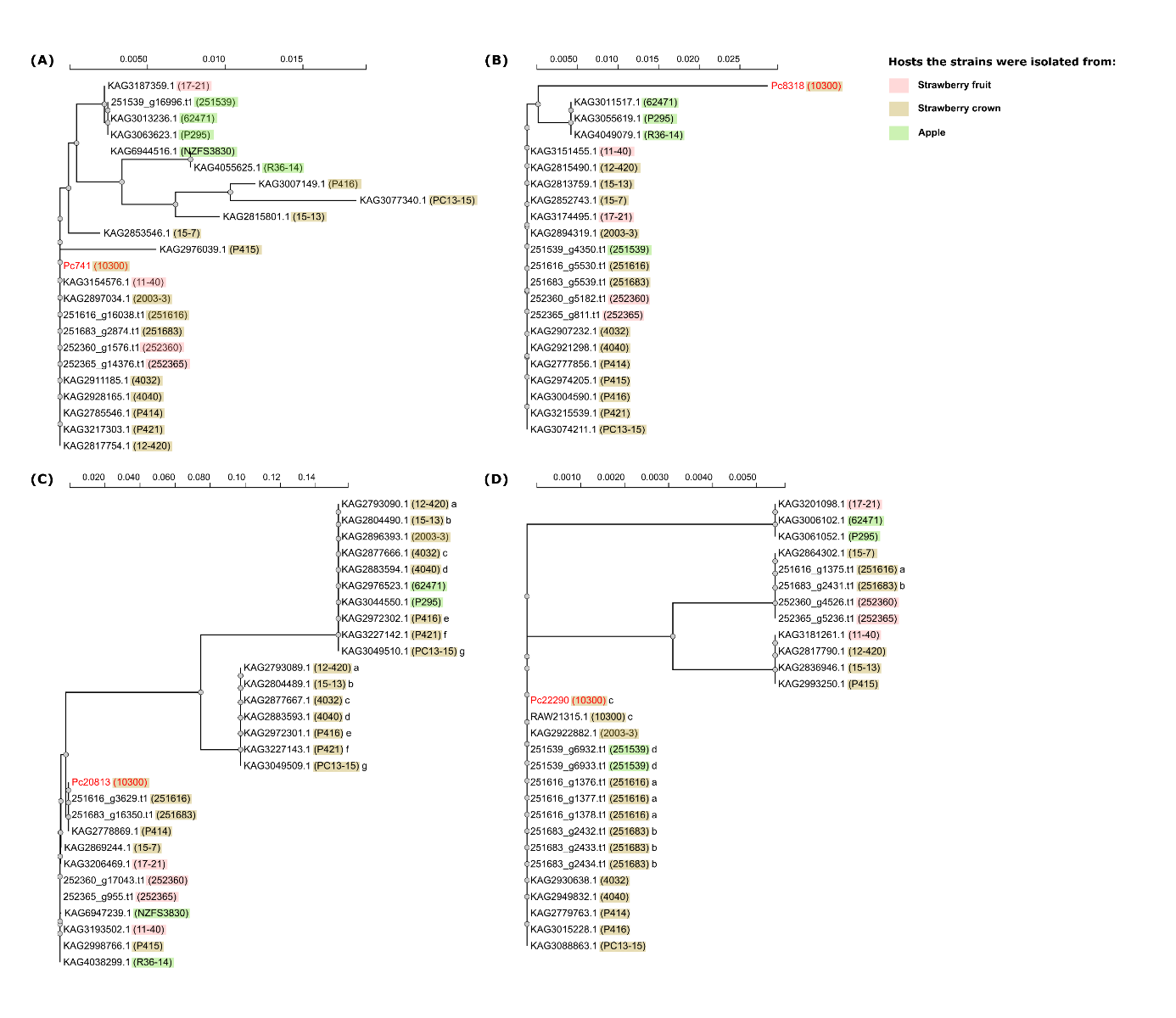
 Supplementary Figure S4. Phylograms of *Phytophthora cactorum* strain 10300 RXLR effectors that induced cell death when transiently expressed in *Nicotiana benthamiana*. (a) Pc741, (b) Pc8318, (c) Pc20813 and (d) Pc22290, are shown in relationship to the respective proteins from 23 different strains of *P. cactorum*. The different strains are given in parenthesis after the proteins with a color code indicating which host or tissue it was isolated from. The RXLR effectors from *P. cactorum* isolate 10300 identified in this study are highlighted in red. The letters a-g after some proteins indicate gene copies or putative paralogous genes from the same strain within a tree. The RXLR effector Pc10890, which also induced cell death in *N. benthamiana* was identical in all available *P. cactorum* genomes and was therefore not included in the phylogenetic analysis.

## Supplementary Tables

Supplementary Table S1. List of primers used in the present study.

| **Primer name**^1^ | **Primer sequences (5´- 3´)**^2^ | **Amplicon**  **size (bp)** | **Annealing**  **Temperature (°C)** | |
| --- | --- | --- | --- | --- |
| Pc741 F | GGGGACAAGTTTGTACAAAAAAGCAGGCTATGCATCGTCCCCAGCTCAAG | 1160 | 59 |  |
| Pc741 R | GGGGACCACTTTGTACAAGAAAGCTGGGTTCAGTCTAAGTTGCCAGCCT |  |  |  |
| RT Pc741 F | CAGGCTGGCAAGAGAGAAG | 146 | 56 |  |
| RT Pc741 R | GAATCCGCGACGACTTTGAG |  |  |  |
| Pc7604 F | GGGGACAAGTTTGTACAAAAAAGCAGGCTATGGCATCAACAGCTTTACGTG | 434 | 57 |  |
| Pc7604 R | GGGGACCACTTTGTACAAGAAAGCTGGGTCTAAATCTGTGGCTTGATCGGTG |  |  |  |
| RT Pc7604 F | GGGACTGGAGATACTTCACG | 140 | 56 |  |
| RT Pc7604 R | GTCAGTCAGTTTCGCTTCTCC |  |  |  |
| Pc7628 F | GGGGACAAGTTTGTACAAAAAAGCAGGCTATGGCATCAGTCGACACGAATAAG | 506 | 59 |  |
| Pc7628 R | GGGGACCACTTTGTACAAGAAAGCTGGGTTTAGTTTTGTTTTGCAAGGTGCTTG |  |  |  |
| RT Pc7628 F | GGTGGAGGCACTCAAACG | 148 | 56 |  |
| RT Pc7628 R | CCACCAGCCACTATACAACAGAG |  |  |  |
| Pc8318 F | GGGGACAAGTTTGTACAAAAAAGCAGGCTATGGCCACCGACTCCGACACG | 1106 | 58 |  |
| Pc8318 R | GGGGACCACTTTGTACAAGAAAGCTGGGTCTACGTAGTTCCGTCCAGCTTG |  |  |  |
| RT Pc8318 F | GTGGGTGGTCAACTTGCTC | 150 | 56 |  |
| RT Pc8318 R | GTCCATCGAAACCCATAAGC |  |  |  |
| Pc8682 F | GGGGACAAGTTTGTACAAAAAAGCAGGCTATGGAGAGCGTCGAAGACCGTC | 470 | 56 |  |
| Pc8682 R | GGGGACCACTTTGTACAAGAAAGCTGGGTTTAAGCCGAACCGGTGGTCGTCG |  |  |  |
| RT Pc8682 F | TGAGGAAGGCCATTACGG | 150 | 56 |  |
| RT Pc8682 R | GCGTAATCTTGGCGAACTTG |  |  |  |
| Pc10890 F | GGGGACAAGTTTGTACAAAAAAGCAGGCTATGGATCAGGCCAGTGTGTTG | 422 | 56 |  |
| Pc10890 R | GGGGACCACTTTGTACAAGAAAGCTGGGTCTATCGTGGTTCGATGATGCG |  |  |  |
| RT Pc10890 F | CGGGTGACGAAGGCAAAATTAC | 130 | 56 |  |
| RT Pc10890 R | CAACGCTGGAACACCTTATCG |  |  |  |
| Pc11254 F | GGGGACAAGTTTGTACAAAAAAGCAGGCTATGAGCTCAGACGCCGAAG | 1043 | 57 |  |
| Pc11254 R | GGGGACCACTTTGTACAAGAAAGCTGGGTTCACTCCAGGGCCTTAATTTTG |  |  |  |
| RT Pc11254 F | AGCACCTGGCTGAAGTATGC | 158 | 56 |  |
| RT Pc11254 R | GCGGCCATTTTCTTAGTCGT |  |  |  |
| Pc12728 F | GGGGACAAGTTTGTACAAAAAAGCAGGCTATGTTATCTGCGTCTGTGGATGTTGATG | 863 | 57 |  |
| Pc12728 R | GGGGACCACTTTGTACAAGAAAGCTGGGTTCAAGTAGTAGCTATCGCCGGCA |  |  |  |
| RT Pc12728 F | TGTCGCTTGGCTGAACAC | 117 | 57 |  |
| RT Pc12728 R | TCAGCGAATGCGGAGTCAG |  |  |  |
| Pc15067 F | GGGGACAAGTTTGTACAAAAAAGCAGGCTATGGCCTACGACTTCGCTAACTC | 470 | 57 |  |
| Pc15067 R | GGGGACCACTTTGTACAAGAAAGCTGGGTTTAGGTCCCAGCGAGAGTG |  |  |  |
| RT Pc15067 F | CTTGATCTCCAAGGTGAAGAACG | 132 | 56 |  |
| RT Pc15067 R | GCAACCCCTCCAATCTCC |  |  |  |
| Pc16451 F | GGGGACAAGTTTGTACAAAAAAGCAGGCTATGGCATCGACTGCAAGTCTGAC | 782 | 56 |  |
| Pc16451 R | GGGGACCACTTTGTACAAGAAAGCTGGGTTGTAGATCAGCGACGGTTGC |  |  |  |
| RT Pc16451 F | CCGCAGAAGCTGAGCAAAC | 126 | 57 |  |
| RT Pc16451 R | GATAGAAGCCTTCGCAGCAG |  |  |  |
| Pc16706 F | GGGGACAAGTTTGTACAAAAAAGCAGGCTATGGCAGAATTCGACCACACTAAAC | 596 | 57 |  |
| Pc16706 R | GGGGACCACTTTGTACAAGAAAGCTGGGTCTAGATACCCAACATGGATCTGAGAC |  |  |  |
| RT Pc16706 F | GGAACGCGCTACTACAACG | 142 | 56 |  |
| RT Pc16706 R | CGAATCAGCTTTCTTTGCCTTC |  |  |  |
| Pc16877 F | GGGGACAAGTTTGTACAAAAAAGCAGGCTATGTCAGTGAATGTCGAACAGTCCAAAC | 395 | 56 |  |
| Pc16877 R | GGGGACCACTTTGTACAAGAAAGCTGGGTTCAGGCCTCCTCTTCATTCTTC |  |  |  |
| RT Pc16877 F | ACGGCTGACCAGAATTCCATC | 146 | 57 |  |
| RT Pc16877 R | GTCCTCCCCAACATGTACCAG |  |  |  |
| Pc17244 F | GGGGACAAGTTTGTACAAAAAAGCAGGCTATGGTTGCTGAGACCTCGAACGG | 389 | 59 |  |
| Pc17244 R | GGGGACCACTTTGTACAAGAAAGCTGGGTTCATGGTGTGGCCAGCCT |  |  |  |
| RT Pc17244 F | AGGATCGCGCAGTATGGAC | 134 | 56 |  |
| RT Pc17244 R | GTAGAGCCGCTGGAGTTG |  |  |  |
| Pc19202 F | GGGGACAAGTTTGTACAAAAAAGCAGGCTATGGCCTCGACGTTCCATCTC | 548 | 56 |  |
| Pc19202 R | GGGGACCACTTTGTACAAGAAAGCTGGGTTCAGGTCTCCACATTCTTTAGACG |  |  |  |
| RT Pc19202 F | GACAAGGCAGCAGGGATG | 136 | 56 |  |
| RT Pc19202 R | CTTGGAGATTGGGCACTAGG |  |  |  |
| Pc20589 F | GGGGACAAGTTTGTACAAAAAAGCAGGCTATGGTGACCATGGATTCCAACCAG | 386 | 56 |  |
| Pc20589 R | GGGGACCACTTTGTACAAGAAAGCTGGGTTTACACTCGCCTTCCAATGTTATG |  |  |  |
| RT Pc20589 F | CTCCGATGACCTCGATGACT | 151 | 56 |  |
| RT Pc20589 R | CTCGTTGAGGACGGCTTG |  |  |  |
| Pc20813 F | GGGGACAAGTTTGTACAAAAAAGCAGGCTATGCTCTCCGCATTTGACTACGAAC | 595 | 56 |  |
| Pc20813 R | GGGGACCACTTTGTACAAGAAAGCTGGGTCATCAATTTTTCGCTGCCAAC |  |  |  |
| RT Pc20813 F | ACCGAAGCGTGGATAAACC | 166 | 56 |  |
| RT Pc20813 R | TGCCTTCGATGCTCATGC |  |  |  |
| Pc21899 F | GGGGACAAGTTTGTACAAAAAAGCAGGCTATGGCCACAGCCTCCGGTCGC | 434 | 56 |  |
| Pc21899 R | GGGGACCACTTTGTACAAGAAAGCTGGGTGGGCCTGCTGTATTAATACGCCCAG |  |  |  |
| RT Pc21899 F | CGAGGAAGAAGACGAAGAAGAC | 120 | 56 |  |
| RT Pc21899 R | TCCAAACCTCCATCATCTCC |  |  |  |
| Pc22014 F | GGGGACAAGTTTGTACAAAAAAGCAGGCTATGAGTGTAACCGTATTAGGCGA | 431 | 56 |  |
| Pc22014 R | GGGGACCACTTTGTACAAGAAAGCTGGGTCTACTTTGGTTCTGGCAGACG |  |  |  |
| RT Pc22014 F | CACCCGAAGAAGATGACGAAG | 151 | 56 |  |
| RT Pc22014 R | GCCTTGCGATAAACGTCATTG |  |  |  |
| Pc22254 F | GGGGACAAGTTTGTACAAAAAAGCAGGCTATGGCGTTCACGAATGCCGAC | 392 | 56 |  |
| Pc22254 R | GGGGACCACTTTGTACAAGAAAGCTGGGTTTAACCTGACTTTGGCAACTTC |  |  |  |
| RT Pc22254 F | CCAGAAGATGCTGATGAAGAG | 150 | 55 |  |
| RT Pc22254 R | CCTAATGGACGCAGCAGT |  |  |  |
| Pc22290 F | GGGGACAAGTTTGTACAAAAAAGCAGGCTATGTTGACGGACTCGAAGCA | 524 | 56 |  |
| Pc22290 R | GGGGACCACTTTGTACAAGAAAGCTGGGTCCAAGATCATTTGTTGCCAGC |  |  |  |
| RT Pc22290 F | ACGCTCGATAAAGTACTCAACGG | 129 | 56 |  |
| RT Pc22290 R | CTTCTTCTCAATGGCTGGGTC |  |  |  |
| EF1α F^3^ | AGCTTTACCTCCCAAGTCATC | 135 | 55 |  |
| EF1α R | AGAACGCCTGTCAATCTTGG |  |  |  |

^1^ Pc in the primer name is the abbreviated form of PC110_g and is obtained from *Phytophthora cactorum* [accession GCA_003287315.1](https://www.ncbi.nlm.nih.gov/assembly/GCA_003287315.1). ‘F’ represents forward primer and ‘R’ represents reverse primer. Primers with RT were used for the reverse transcription PCR.

^2^ The underscores in the forward and reverse primer sequences represent attB1 and attB2 sites, respectively.

^3^ Sequences of primer pair obtained from Liu *et al.* (2012)
